# Analysis of the Menstrual Fluid

**Published:** 1843-01

**Authors:** 


*Analysis of the Menstrual Fluid.*—Bouchardat undertook a new analysis of the menstrual fluid, obtained from a female, who consented to allow a speculum to remain in her vagina for ten hours, in order that an ounce of it might be procured. Without this precaution the fluid becomes mixed with vaginal mucus and urine, as the presence of ammoniaco-magnesian phosphate proves. The following is the analysis: water, 90.8; fixed matters, 6.92. The fixed matters were thus composed; fibrine, albumen, and colouring matter, 75.27; extractive matter, 0.42; fatty matter, 2.21; salts, 5.31; mucus, 16.79. The female from whom the secretion was obtained was a patient of Boismont's, and he considers the great proportion of water due to the delicacy of her frame, and her subsisting chiefly on a vegetable diet. Another specimen of menstrual fluid, examined by Donné, gave the following microscopic characters: 1st, abundance of the ordinary globules of the blood; 2d, vaginal mucus formed of epidermic squamæ from the mucous membrane of the vagina; 3d, mucous globules furnished by the neck of the uterus. From these examinations it results that the menstrual fluid does not differ from arterial blood. As to the acid or alkaline nature of the fluid, observed by authors, it depends upon the presence of mucus from the vagina and neck of the uterus. This mucus, as Nauche has proved, is acid in a healthy woman and after delivery, but becomes alkaline when it is glairous, or the product of inflammation; if only a limited portion of the passage be affected, the secretion will be acid in one part and alkaline in another.—*Provi. Med. Journ.*, July 30, 1842.

